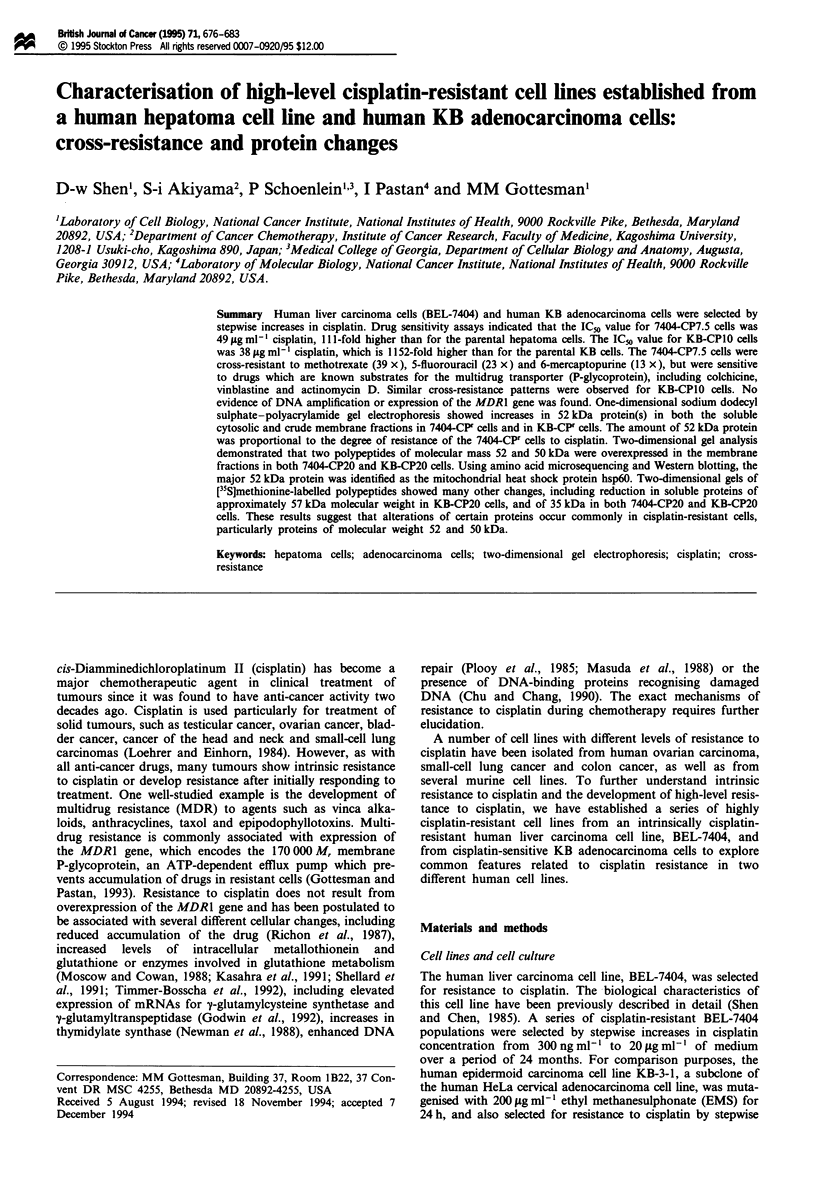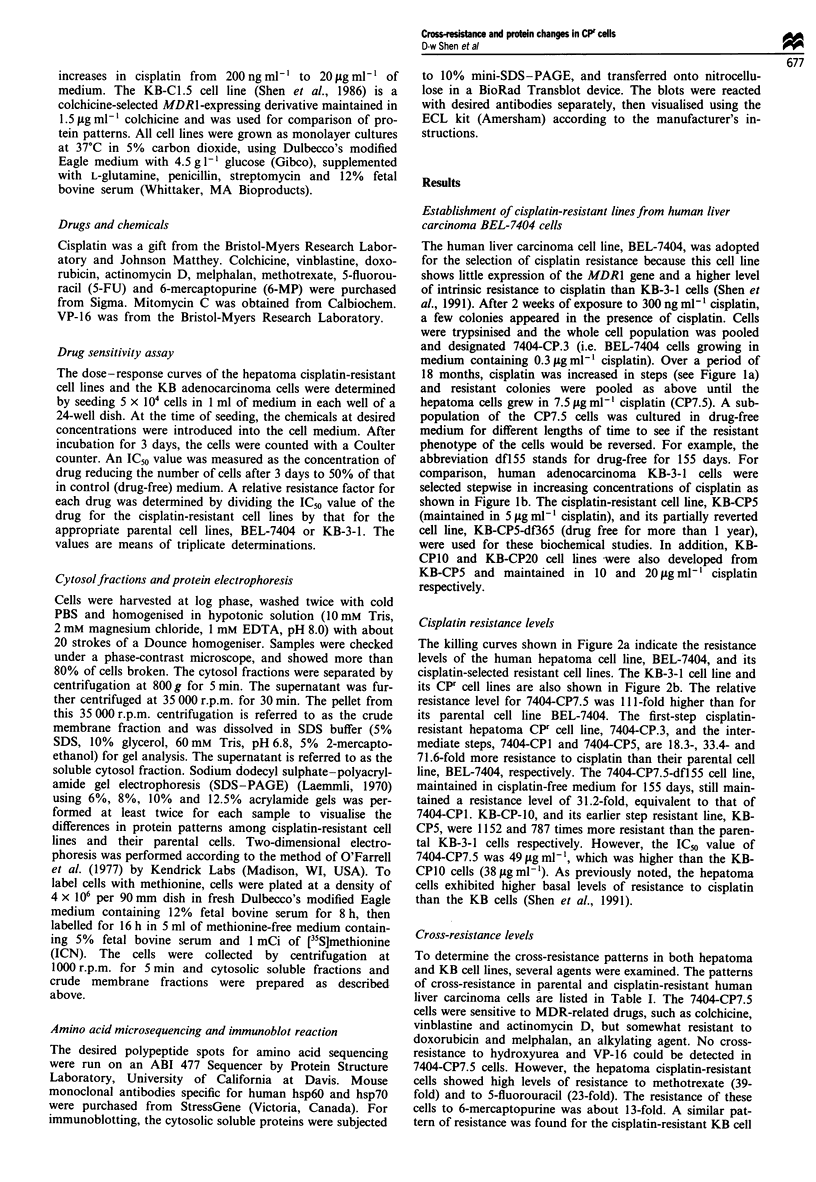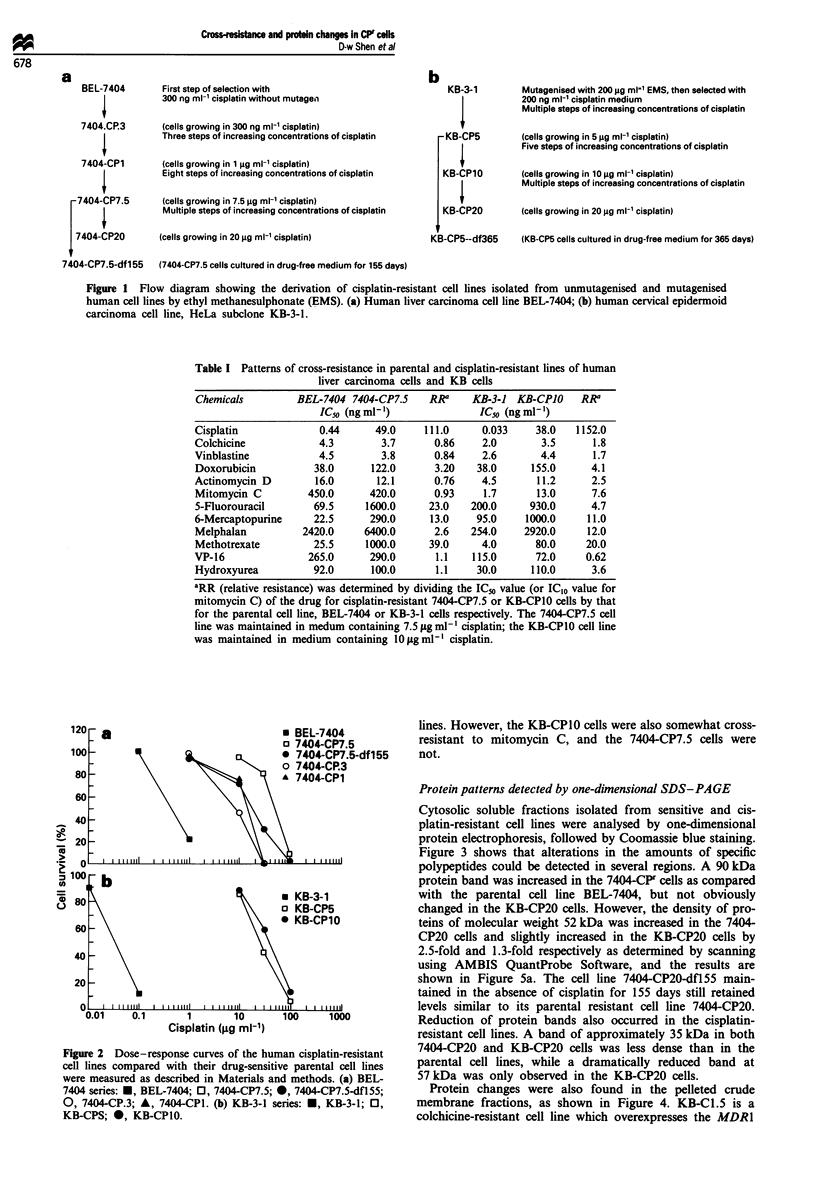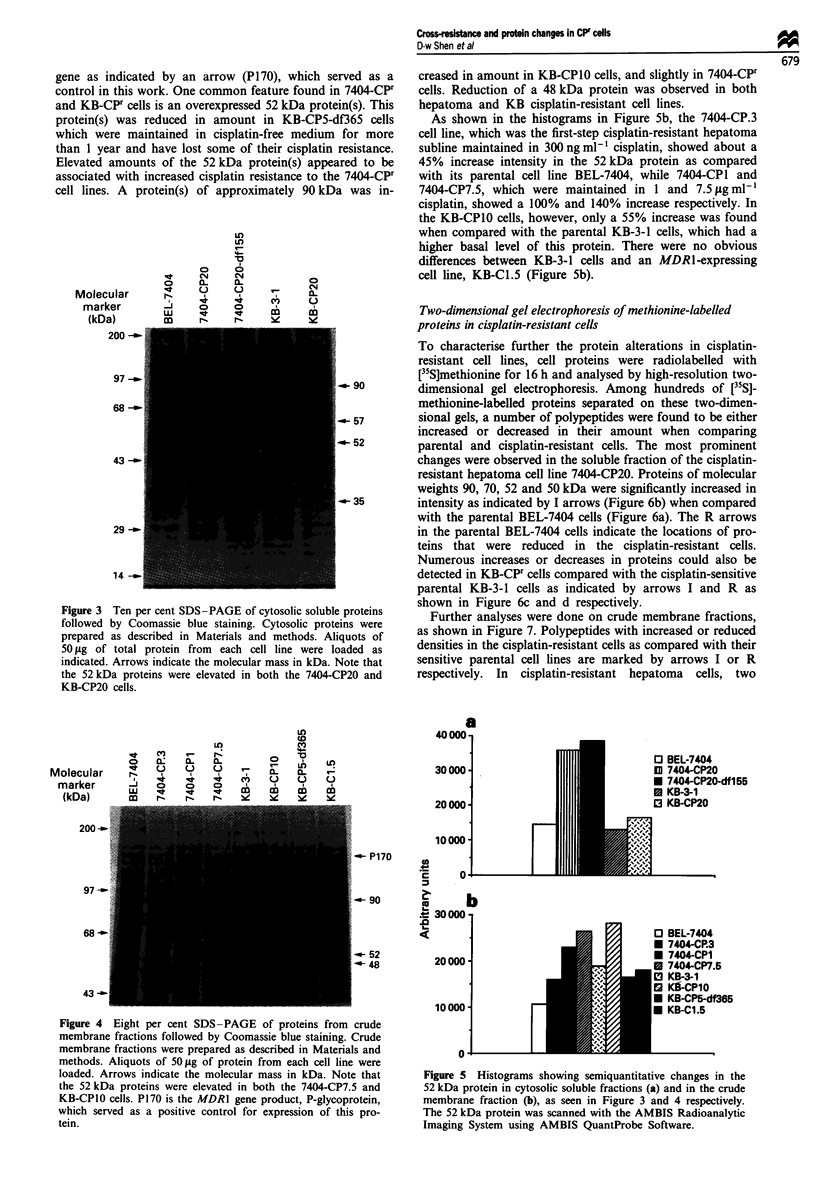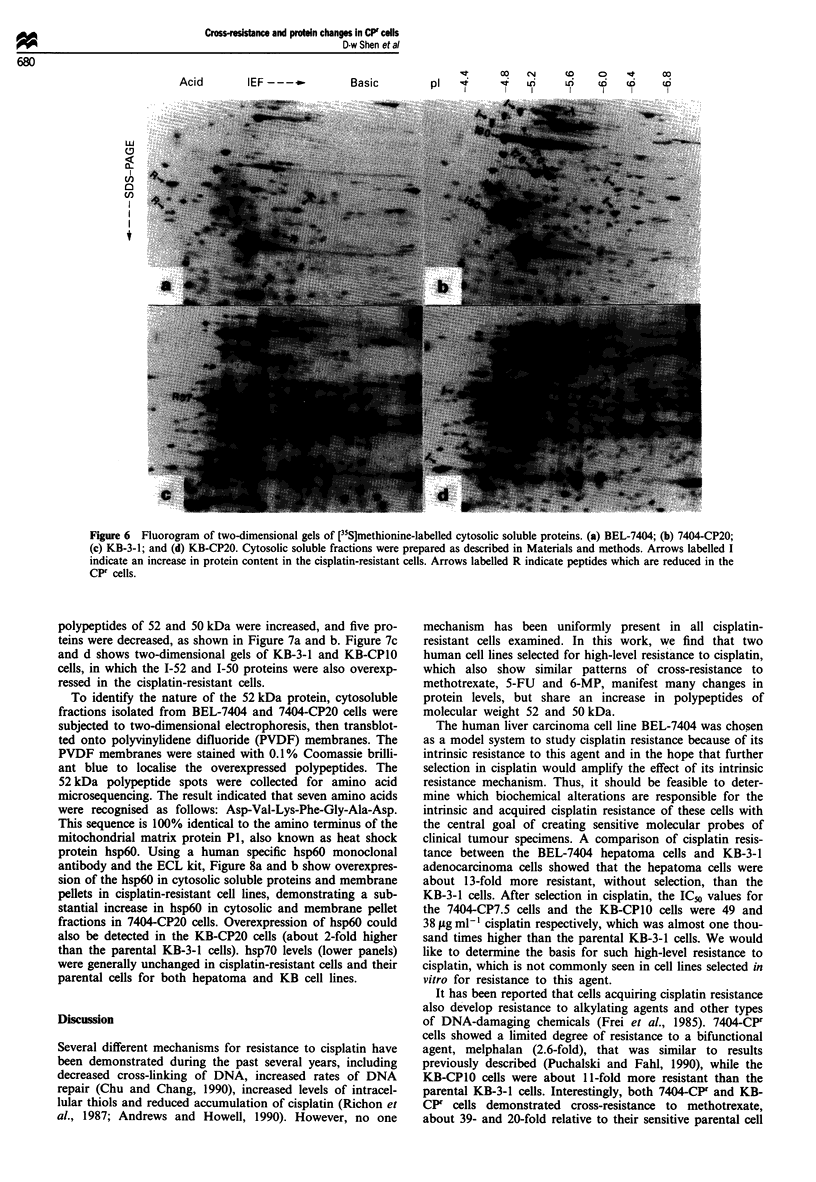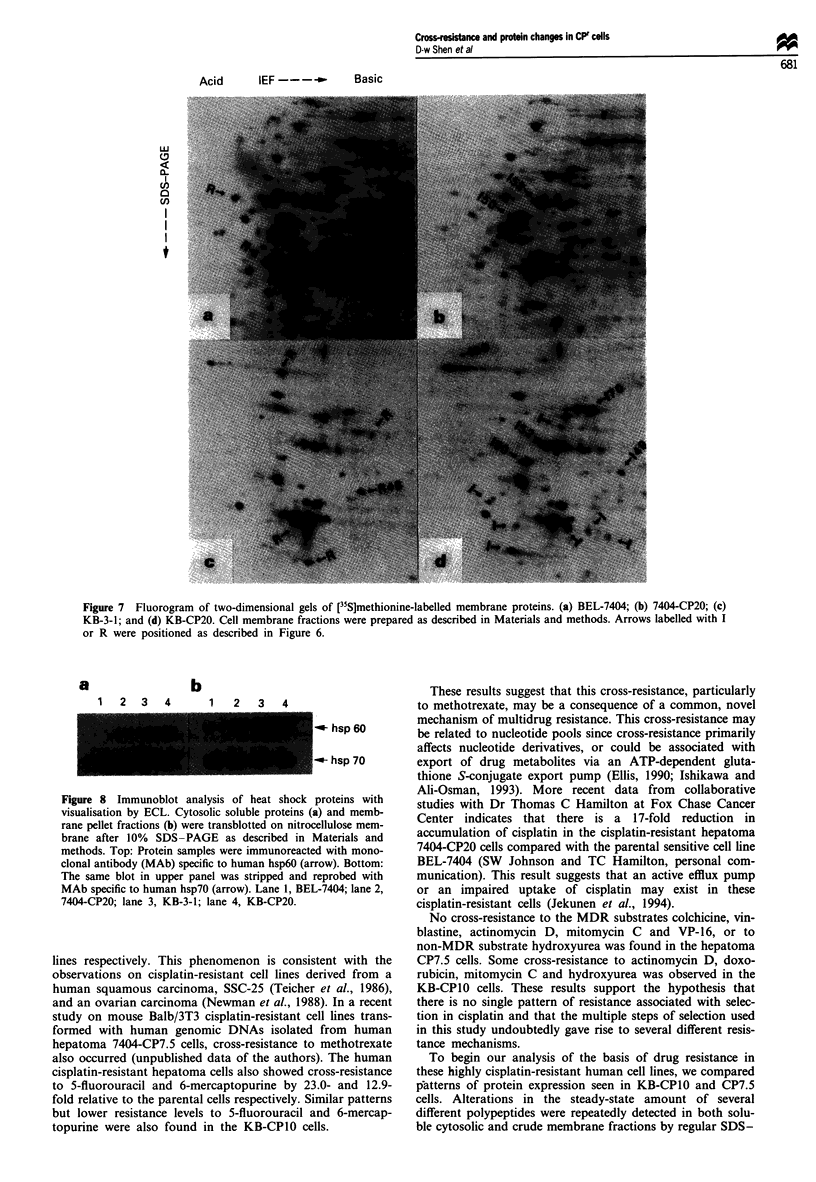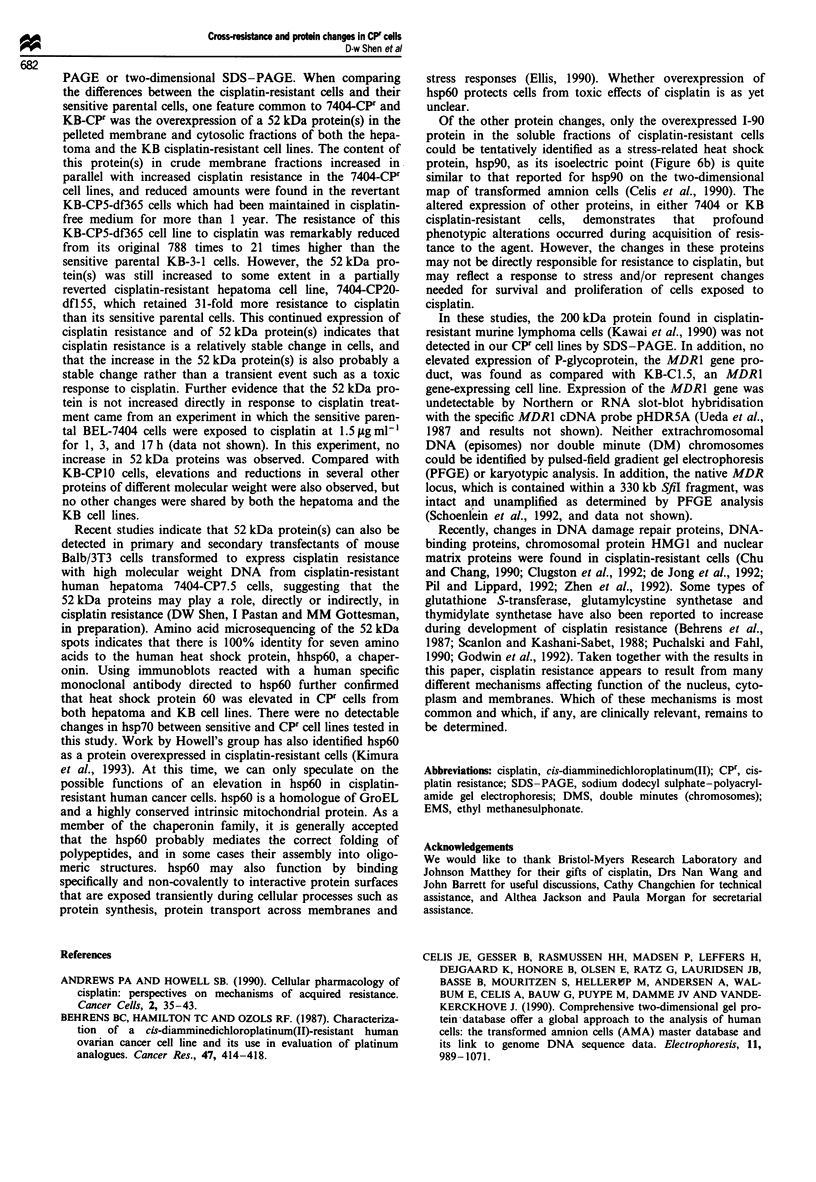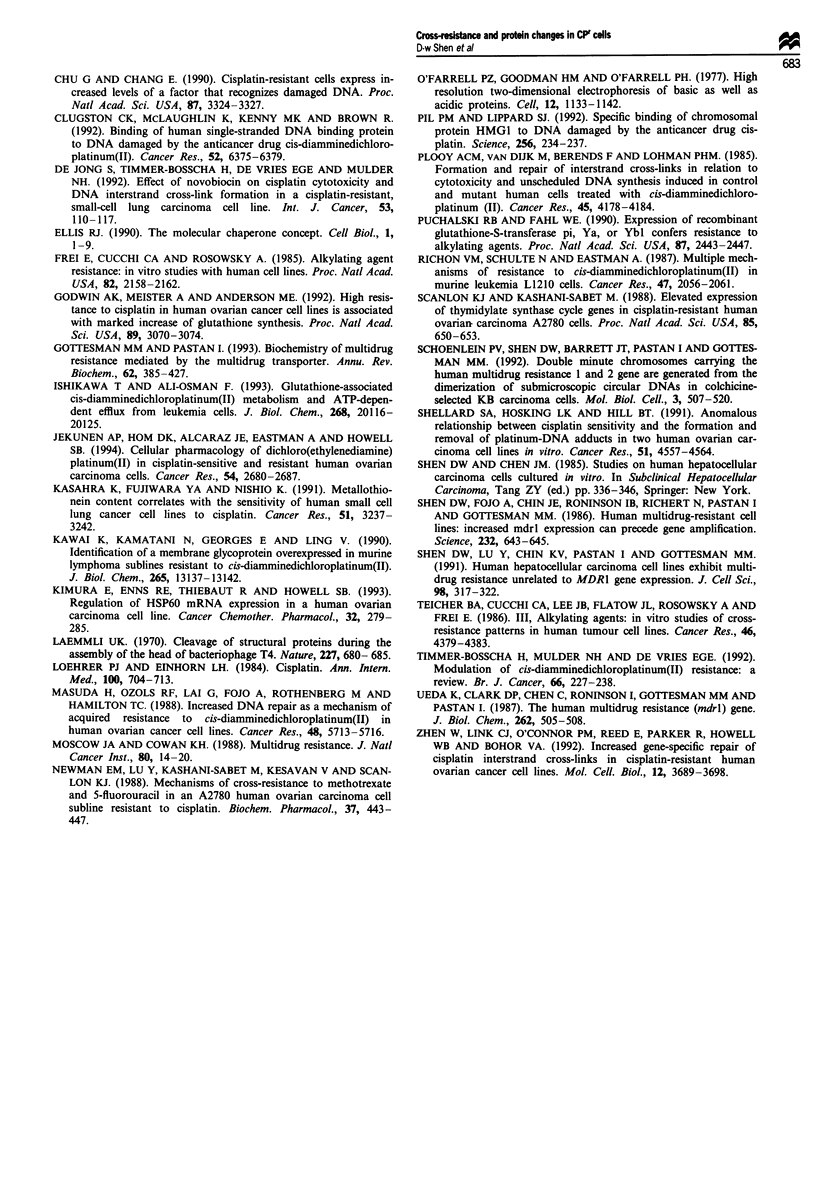# Characterisation of high-level cisplatin-resistant cell lines established from a human hepatoma cell line and human KB adenocarcinoma cells: cross-resistance and protein changes.

**DOI:** 10.1038/bjc.1995.134

**Published:** 1995-04

**Authors:** D. W. Shen, S. Akiyama, P. Schoenlein, I. Pastan, M. M. Gottesman

**Affiliations:** Laboratory of Cell Biology, National Cancer Institute, National Institutes of Health, Bethesda, Maryland 20892, USA.

## Abstract

**Images:**


British Journal of Cancer (1995) 71, 676-683
© 1995 Stockton Press All rights reserved 0007-0920/95 $12.00

### Characterisation of high-level cisplatin-resistant cell lines established from a human hepatoma cell line and human KB adenocarcinoma cells: cross-resistance and protein changes

D-w Shen[1], S-i Akiyama[2], P Schoenlein[1,3], I Pastan[4] and MM Gottesman[1]

*[1]Laboratory of Cell Biology, National Cancer Institute, National Institutes of Health, 9000 Rockville Pike, Bethesda, Maryland 20892, USA; [2]Department of Cancer Chemotherapy, Institute of Cancer Research, Faculty of Medicine, Kagoshima University, 1208-1 Usuki-cho, Kagoshima 890, Japan; [3]Medical College of Georgia, Department of Cellular Biology and Anatomy, Augusta, Georgia 30912, USA; [4]Laboratory of Molecular Biology, National Cancer Institute, National Institutes of Health, 9000 Rockville Pike, Bethesda, Maryland 20892, USA.*

**Summary** Human liver carcinoma cells (BEL-7404) and human KB adenocarcinoma cells were selected by stepwise increases in cisplatin. Drug sensitivity assays indicated that the $IC_{50}$ value for 7404-CP7.5 cells was 49 $\mu g\ ml^{-1}$ cisplatin, 111-fold higher than for the parental hepatoma cells. The $IC_{50}$ value for KB-CP10 cells was 38 $\mu g\ ml^{-1}$ cisplatin, which is 1152-fold higher than for the parental KB cells. The 7404-CP7.5 cells were cross-resistant to methotrexate (39 ×), 5-fluorouracil (23 ×) and 6-mercaptopurine (13 ×), but were sensitive to drugs which are known substrates for the multidrug transporter (P-glycoprotein), including colchicine, vinblastine and actinomycin D. Similar cross-resistance patterns were observed for KB-CP10 cells. No evidence of DNA amplification or expression of the *MDR*1 gene was found. One-dimensional sodium dodecyl sulphate–polyacrylamide gel electrophoresis showed increases in 52 kDa protein(s) in both the soluble cytosolic and crude membrane fractions in 7404-$CP^r$ cells and in KB-$CP^r$ cells. The amount of 52 kDa protein was proportional to the degree of resistance of the 7404-$CP^r$ cells to cisplatin. Two-dimensional gel analysis demonstrated that two polypeptides of molecular mass 52 and 50 kDa were overexpressed in the membrane fractions in both 7404-CP20 and KB-CP20 cells. Using amino acid microsequencing and Western blotting, the major 52 kDa protein was identified as the mitochondrial heat shock protein hsp60. Two-dimensional gels of [$^{35}$S]methionine-labelled polypeptides showed many other changes, including reduction in soluble proteins of approximately 57 kDa molecular weight in KB-CP20 cells, and of 35 kDa in both 7404-CP20 and KB-CP20 cells. These results suggest that alterations of certain proteins occur commonly in cisplatin-resistant cells, particularly proteins of molecular weight 52 and 50 kDa.

**Keywords:** hepatoma cells; adenocarcinoma cells; two-dimensional gel electrophoresis; cisplatin; cross-resistance

*cis*-Diamminedichloroplatinum II (cisplatin) has become a major chemotherapeutic agent in clinical treatment of tumours since it was found to have anti-cancer activity two decades ago. Cisplatin is used particularly for treatment of solid tumours, such as testicular cancer, ovarian cancer, bladder cancer, cancer of the head and neck and small-cell lung carcinomas (Loehrer and Einhorn, 1984). However, as with all anti-cancer drugs, many tumours show intrinsic resistance to cisplatin or develop resistance after initially responding to treatment. One well-studied example is the development of multidrug resistance (MDR) to agents such as vinca alkaloids, anthracyclines, taxol and epipodophyllotoxins. Multidrug resistance is commonly associated with expression of the *MDR*1 gene, which encodes the 170 000 $M_r$ membrane P-glycoprotein, an ATP-dependent efflux pump which prevents accumulation of drugs in resistant cells (Gottesman and Pastan, 1993). Resistance to cisplatin does not result from overexpression of the *MDR*1 gene and has been postulated to be associated with several different cellular changes, including reduced accumulation of the drug (Richon *et al.*, 1987), increased levels of intracellular metallothionein and glutathione or enzymes involved in glutathione metabolism (Moscow and Cowan, 1988; Kasahra *et al.*, 1991; Shellard *et al.*, 1991; Timmer-Bosscha *et al.*, 1992), including elevated expression of mRNAs for γ-glutamylcysteine synthetase and γ-glutamyltranspeptidase (Godwin *et al.*, 1992), increases in thymidylate synthase (Newman *et al.*, 1988), enhanced DNA repair (Plooy *et al.*, 1985; Masuda *et al.*, 1988) or the presence of DNA-binding proteins recognising damaged DNA (Chu and Chang, 1990). The exact mechanisms of resistance to cisplatin during chemotherapy requires further elucidation.

A number of cell lines with different levels of resistance to cisplatin have been isolated from human ovarian carcinoma, small-cell lung cancer and colon cancer, as well as from several murine cell lines. To further understand intrinsic resistance to cisplatin and the development of high-level resistance to cisplatin, we have established a series of highly cisplatin-resistant cell lines from an intrinsically cisplatin-resistant human liver carcinoma cell line, BEL-7404, and from cisplatin-sensitive KB adenocarcinoma cells to explore common features related to cisplatin resistance in two different human cell lines.

### Materials and methods

### *Cell lines and cell culture*

The human liver carcinoma cell line, BEL-7404, was selected for resistance to cisplatin. The biological characteristics of this cell line have been previously described in detail (Shen and Chen, 1985). A series of cisplatin-resistant BEL-7404 populations were selected by stepwise increases in cisplatin concentration from 300 $ng\ ml^{-1}$ to 20 $\mu g\ ml^{-1}$ of medium over a period of 24 months. For comparison purposes, the human epidermoid carcinoma cell line KB-3-1, a subclone of the human HeLa cervical adenocarcinoma cell line, was mutagenised with 200 $\mu g\ ml^{-1}$ ethyl methanesulphonate (EMS) for 24 h, and also selected for resistance to cisplatin by stepwise

Correspondence: MM Gottesman, Building 37, Room 1B22, 37 Convent DR MSC 4255, Bethesda MD 20892-4255, USA
Received 5 August 1994; revised 18 November 1994; accepted 7 December 1994

increases in cisplatin from 200 ng ml$^{-1}$ to 20 $\mu$g ml$^{-1}$ of medium. The KB-C1.5 cell line (Shen *et al.*, 1986) is a colchicine-selected *MDR*1-expressing derivative maintained in 1.5 $\mu$g ml$^{-1}$ colchicine and was used for comparison of protein patterns. All cell lines were grown as monolayer cultures at 37°C in 5% carbon dioxide, using Dulbecco's modified Eagle medium with 4.5 g l$^{-1}$ glucose (Gibco), supplemented with L-glutamine, penicillin, streptomycin and 12% fetal bovine serum (Whittaker, MA Bioproducts).

### *Drugs and chemicals*

Cisplatin was a gift from the Bristol-Myers Research Laboratory and Johnson Matthey. Colchicine, vinblastine, doxorubicin, actinomycin D, melphalan, methotrexate, 5-fluorouracil (5-FU) and 6-mercaptopurine (6-MP) were purchased from Sigma. Mitomycin C was obtained from Calbiochem. VP-16 was from the Bristol-Myers Research Laboratory.

### *Drug sensitivity assay*

The dose-response curves of the hepatoma cisplatin-resistant cell lines and the KB adenocarcinoma cells were determined by seeding $5 \times 10^4$ cells in 1 ml of medium in each well of a 24-well dish. At the time of seeding, the chemicals at desired concentrations were introduced into the cell medium. After incubation for 3 days, the cells were counted with a Coulter counter. An $IC_{50}$ value was measured as the concentration of drug reducing the number of cells after 3 days to 50% of that in control (drug-free) medium. A relative resistance factor for each drug was determined by dividing the $IC_{50}$ value of the drug for the cisplatin-resistant cell lines by that for the appropriate parental cell lines, BEL-7404 or KB-3-1. The values are means of triplicate determinations.

### *Cytosol fractions and protein electrophoresis*

Cells were harvested at log phase, washed twice with cold PBS and homogenised in hypotonic solution (10 mM Tris, 2 mM magnesium chloride, 1 mM EDTA, pH 8.0) with about 20 strokes of a Dounce homogeniser. Samples were checked under a phase-contrast microscope, and showed more than 80% of cells broken. The cytosol fractions were separated by centrifugation at 800 *g* for 5 min. The supernatant was further centrifuged at 35 000 r.p.m. for 30 min. The pellet from this 35 000 r.p.m. centrifugation is referred to as the crude membrane fraction and was dissolved in SDS buffer (5% SDS, 10% glycerol, 60 mM Tris, pH 6.8, 5% 2-mercaptoethanol) for gel analysis. The supernatant is referred to as the soluble cytosol fraction. Sodium dodecyl sulphate-polyacrylamide gel electrophoresis (SDS-PAGE) (Laemmli, 1970) using 6%, 8%, 10% and 12.5% acrylamide gels was performed at least twice for each sample to visualise the differences in protein patterns among cisplatin-resistant cell lines and their parental cells. Two-dimensional electrophoresis was performed according to the method of O'Farrell *et al.* (1977) by Kendrick Labs (Madison, WI, USA). To label cells with methionine, cells were plated at a density of $4 \times 10^6$ per 90 mm dish in fresh Dulbecco's modified Eagle medium containing 12% fetal bovine serum for 8 h, then labelled for 16 h in 5 ml of methionine-free medium containing 5% fetal bovine serum and 1 mCi of [$^{35}$S]methionine (ICN). The cells were collected by centrifugation at 1000 r.p.m. for 5 min and cytosolic soluble fractions and crude membrane fractions were prepared as described above.

### *Amino acid microsequencing and immunoblot reaction*

The desired polypeptide spots for amino acid sequencing were run on an ABI 477 Sequencer by Protein Structure Laboratory, University of California at Davis. Mouse monoclonal antibodies specific for human hsp60 and hsp70 were purchased from StressGene (Victoria, Canada). For immunoblotting, the cytosolic soluble proteins were subjected to 10% mini-SDS-PAGE, and transferred onto nitrocellulose in a BioRad Transblot device. The blots were reacted with desired antibodies separately, then visualised using the ECL kit (Amersham) according to the manufacturer's instructions.

### Results

### *Establishment of cisplatin-resistant lines from human liver carcinoma BEL-7404 cells*

The human liver carcinoma cell line, BEL-7404, was adopted for the selection of cisplatin resistance because this cell line shows little expression of the *MDR*1 gene and a higher level of intrinsic resistance to cisplatin than KB-3-1 cells (Shen *et al.*, 1991). After 2 weeks of exposure to 300 ng ml$^{-1}$ cisplatin, a few colonies appeared in the presence of cisplatin. Cells were trypsinised and the whole cell population was pooled and designated 7404-CP.3 (i.e. BEL-7404 cells growing in medium containing 0.3 $\mu$g ml$^{-1}$ cisplatin). Over a period of 18 months, cisplatin was increased in steps (see Figure 1a) and resistant colonies were pooled as above until the hepatoma cells grew in 7.5 $\mu$g ml$^{-1}$ cisplatin (CP7.5). A subpopulation of the CP7.5 cells was cultured in drug-free medium for different lengths of time to see if the resistant phenotype of the cells would be reversed. For example, the abbreviation df155 stands for drug-free for 155 days. For comparison, human adenocarcinoma KB-3-1 cells were selected stepwise in increasing concentrations of cisplatin as shown in Figure 1b. The cisplatin-resistant cell line, KB-CP5 (maintained in 5 $\mu$g ml$^{-1}$ cisplatin), and its partially reverted cell line, KB-CP5-df365 (drug free for more than 1 year), were used for these biochemical studies. In addition, KB-CP10 and KB-CP20 cell lines were also developed from KB-CP5 and maintained in 10 and 20 $\mu$g ml$^{-1}$ cisplatin respectively.

### *Cisplatin resistance levels*

The killing curves shown in Figure 2a indicate the resistance levels of the human hepatoma cell line, BEL-7404, and its cisplatin-selected resistant cell lines. The KB-3-1 cell line and its CP$^r$ cell lines are also shown in Figure 2b. The relative resistance level for 7404-CP7.5 was 111-fold higher than for its parental cell line BEL-7404. The first-step cisplatin-resistant hepatoma CP$^r$ cell line, 7404-CP.3, and the intermediate steps, 7404-CP1 and 7404-CP5, are 18.3-, 33.4- and 71.6-fold more resistance to cisplatin than their parental cell line, BEL-7404, respectively. The 7404-CP7.5-df155 cell line, maintained in cisplatin-free medium for 155 days, still maintained a resistance level of 31.2-fold, equivalent to that of 7404-CP1. KB-CP-10, and its earlier step resistant line, KB-CP5, were 1152 and 787 times more resistant than the parental KB-3-1 cells respectively. However, the $IC_{50}$ value of 7404-CP7.5 was 49 $\mu$g ml$^{-1}$, which was higher than the KB-CP10 cells (38 $\mu$g ml$^{-1}$). As previously noted, the hepatoma cells exhibited higher basal levels of resistance to cisplatin than the KB cells (Shen *et al.*, 1991).

### *Cross-resistance levels*

To determine the cross-resistance patterns in both hepatoma and KB cell lines, several agents were examined. The patterns of cross-resistance in parental and cisplatin-resistant human liver carcinoma cells are listed in Table I. The 7404-CP7.5 cells were sensitive to MDR-related drugs, such as colchicine, vinblastine and actinomycin D, but somewhat resistant to doxorubicin and melphalan, an alkylating agent. No cross-resistance to hydroxyurea and VP-16 could be detected in 7404-CP7.5 cells. However, the hepatoma cisplatin-resistant cells showed high levels of resistance to methotrexate (39-fold) and to 5-fluorouracil (23-fold). The resistance of these cells to 6-mercaptopurine was about 13-fold. A similar pattern of resistance was found for the cisplatin-resistant KB cell

**Figure 1** Flow diagram showing the derivation of cisplatin-resistant cell lines isolated from unmutagenised and mutagenised human cell lines by ethyl methanesulphonate (EMS). (**a**) Human liver carcinoma cell line BEL-7404; (**b**) human cervical epidermoid carcinoma cell line, HeLa subclone KB-3-1.

**Table I** Patterns of cross-resistance in parental and cisplatin-resistant lines of human liver carcinoma cells and KB cells

| *Chemicals* | *BEL-7404* $IC_{50}$ (ng ml$^{-1}$) | *7404-CP7.5* | *RR*[a] | *KB-3-1* $IC_{50}$ (ng ml$^{-1}$) | *KB-CP10* | *RR*[a] |
|---|---|---|---|---|---|---|
| Cisplatin | 0.44 | 49.0 | 111.0 | 0.033 | 38.0 | 1152.0 |
| Colchicine | 4.3 | 3.7 | 0.86 | 2.0 | 3.5 | 1.8 |
| Vinblastine | 4.5 | 3.8 | 0.84 | 2.6 | 4.4 | 1.7 |
| Doxorubicin | 38.0 | 122.0 | 3.20 | 38.0 | 155.0 | 4.1 |
| Actinomycin D | 16.0 | 12.1 | 0.76 | 4.5 | 11.2 | 2.5 |
| Mitomycin C | 450.0 | 420.0 | 0.93 | 1.7 | 13.0 | 7.6 |
| 5-Fluorouracil | 69.5 | 1600.0 | 23.0 | 200.0 | 930.0 | 4.7 |
| 6-Mercaptopurine | 22.5 | 290.0 | 13.0 | 95.0 | 1000.0 | 11.0 |
| Melphalan | 2420.0 | 6400.0 | 2.6 | 254.0 | 2920.0 | 12.0 |
| Methotrexate | 25.5 | 1000.0 | 39.0 | 4.0 | 80.0 | 20.0 |
| VP-16 | 265.0 | 290.0 | 1.1 | 115.0 | 72.0 | 0.62 |
| Hydroxyurea | 92.0 | 100.0 | 1.1 | 30.0 | 110.0 | 3.6 |

[a]RR (relative resistance) was determined by dividing the $IC_{50}$ value (or $IC_{10}$ value for mitomycin C) of the drug for cisplatin-resistant 7404-CP7.5 or KB-CP10 cells by that for the parental cell line, BEL-7404 or KB-3-1 cells respectively. The 7404-CP7.5 cell line was maintained in medum containing 7.5 µg ml$^{-1}$ cisplatin; the KB-CP10 cell line was maintained in medium containing 10 µg ml$^{-1}$ cisplatin.

**Figure 2** Dose–response curves of the human cisplatin-resistant cell lines compared with their drug-sensitive parental cell lines were measured as described in Materials and methods. (**a**) BEL-7404 series: ■, BEL-7404; □, 7404-CP7.5; ●, 7404-CP7.5-df155; O, 7404-CP.3; ▲, 7404-CP1. (**b**) KB-3-1 series: ■, KB-3-1; □, KB-CPS; ●, KB-CP10.

lines. However, the KB-CP10 cells were also somewhat cross-resistant to mitomycin C, and the 7404-CP7.5 cells were not.

### *Protein patterns detected by one-dimensional SDS–PAGE*

Cytosolic soluble fractions isolated from sensitive and cisplatin-resistant cell lines were analysed by one-dimensional protein electrophoresis, followed by Coomassie blue staining. Figure 3 shows that alterations in the amounts of specific polypeptides could be detected in several regions. A 90 kDa protein band was increased in the 7404-CP[r] cells as compared with the parental cell line BEL-7404, but not obviously changed in the KB-CP20 cells. However, the density of proteins of molecular weight 52 kDa was increased in the 7404-CP20 cells and slightly increased in the KB-CP20 cells by 2.5-fold and 1.3-fold respectively as determined by scanning using AMBIS QuantProbe Software, and the results are shown in Figure 5a. The cell line 7404-CP20-df155 maintained in the absence of cisplatin for 155 days still retained levels similar to its parental resistant cell line 7404-CP20. Reduction of protein bands also occurred in the cisplatin-resistant cell lines. A band of approximately 35 kDa in both 7404-CP20 and KB-CP20 cells was less dense than in the parental cell lines, while a dramatically reduced band at 57 kDa was only observed in the KB-CP20 cells.

Protein changes were also found in the pelleted crude membrane fractions, as shown in Figure 4. KB-C1.5 is a colchicine-resistant cell line which overexpresses the *MDR*1

gene as indicated by an arrow (P170), which served as a control in this work. One common feature found in 7404-CP^r and KB-CP^r cells is an overexpressed 52 kDa protein(s). This protein(s) was reduced in amount in KB-CP5-df365 cells which were maintained in cisplatin-free medium for more than 1 year and have lost some of their cisplatin resistance. Elevated amounts of the 52 kDa protein(s) appeared to be associated with increased cisplatin resistance to the 7404-CP^r cell lines. A protein(s) of approximately 90 kDa was increased in amount in KB-CP10 cells, and slightly in 7404-CP^r cells. Reduction of a 48 kDa protein was observed in both hepatoma and KB cisplatin-resistant cell lines.

As shown in the histograms in Figure 5b, the 7404-CP.3 cell line, which was the first-step cisplatin-resistant hepatoma subline maintained in 300 ng $ml^{-1}$ cisplatin, showed about a 45% increase intensity in the 52 kDa protein as compared with its parental cell line BEL-7404, while 7404-CP1 and 7404-CP7.5, which were maintained in 1 and 7.5 $\mu g\ ml^{-1}$ cisplatin, showed a 100% and 140% increase respectively. In the KB-CP10 cells, however, only a 55% increase was found when compared with the parental KB-3-1 cells, which had a higher basal level of this protein. There were no obvious differences between KB-3-1 cells and an *MDR*1-expressing cell line, KB-C1.5 (Figure 5b).

**Figure 3** Ten per cent SDS-PAGE of cytosolic soluble proteins followed by Coomassie blue staining. Cytosolic proteins were prepared as described in Materials and methods. Aliquots of 50 μg of total protein from each cell line were loaded as indicated. Arrows indicate the molecular mass in kDa. Note that the 52 kDa proteins were elevated in both the 7404-CP20 and KB-CP20 cells.

### *Two-dimensional gel electrophoresis of methionine-labelled proteins in cisplatin-resistant cells*

To characterise further the protein alterations in cisplatin-resistant cell lines, cell proteins were radiolabelled with [$^{35}$S]methionine for 16 h and analysed by high-resolution two-dimensional gel electrophoresis. Among hundreds of [$^{35}$S]-methionine-labelled proteins separated on these two-dimensional gels, a number of polypeptides were found to be either increased or decreased in their amount when comparing parental and cisplatin-resistant cells. The most prominent changes were observed in the soluble fraction of the cisplatin-resistant hepatoma cell line 7404-CP20. Proteins of molecular weights 90, 70, 52 and 50 kDa were significantly increased in intensity as indicated by I arrows (Figure 6b) when compared with the parental BEL-7404 cells (Figure 6a). The R arrows in the parental BEL-7404 cells indicate the locations of proteins that were reduced in the cisplatin-resistant cells. Numerous increases or decreases in proteins could also be detected in KB-CP^r cells compared with the cisplatin-sensitive parental KB-3-1 cells as indicated by arrows I and R as shown in Figure 6c and d respectively.

Further analyses were done on crude membrane fractions, as shown in Figure 7. Polypeptides with increased or reduced densities in the cisplatin-resistant cells as compared with their sensitive parental cell lines are marked by arrows I or R respectively. In cisplatin-resistant hepatoma cells, two

**Figure 4** Eight per cent SDS-PAGE of proteins from crude membrane fractions followed by Coomassie blue staining. Crude membrane fractions were prepared as described in Materials and methods. Aliquots of 50 μg of protein from each cell line were loaded. Arrows indicate the molecular mass in kDa. Note that the 52 kDa proteins were elevated in both the 7404-CP7.5 and KB-CP10 cells. P170 is the *MDR*1 gene product, P-glycoprotein, which served as a positive control for expression of this protein.

**Figure 5** Histograms showing semiquantitative changes in the 52 kDa protein in cytosolic soluble fractions (**a**) and in the crude membrane fraction (**b**), as seen in Figure 3 and 4 respectively. The 52 kDa protein was scanned with the AMBIS Radioanalytic Imaging System using AMBIS QuantProbe Software.

**Figure 6** Fluorogram of two-dimensional gels of [$^{35}$S]methionine-labelled cytosolic soluble proteins. (**a**) BEL-7404; (**b**) 7404-CP20; (**c**) KB-3-1; and (**d**) KB-CP20. Cytosolic soluble fractions were prepared as described in Materials and methods. Arrows labelled I indicate an increase in protein content in the cisplatin-resistant cells. Arrows labelled R indicate peptides which are reduced in the CP$^r$ cells.

polypeptides of 52 and 50 kDa were increased, and five proteins were decreased, as shown in Figure 7a and b. Figure 7c and d shows two-dimensional gels of KB-3-1 and KB-CP10 cells, in which the I-52 and I-50 proteins were also overexpressed in the cisplatin-resistant cells.

To identify the nature of the 52 kDa protein, cytosoluble fractions isolated from BEL-7404 and 7404-CP20 cells were subjected to two-dimensional electrophoresis, then transblotted onto polyvinylidene difluoride (PVDF) membranes. The PVDF membranes were stained with 0.1% Coomassie brilliant blue to localise the overexpressed polypeptides. The 52 kDa polypeptide spots were collected for amino acid microsequencing. The result indicated that seven amino acids were recognised as follows: Asp-Val-Lys-Phe-Gly-Ala-Asp. This sequence is 100% identical to the amino terminus of the mitochondrial matrix protein P1, also known as heat shock protein hsp60. Using a human specific hsp60 monoclonal antibody and the ECL kit, Figure 8a and b show overexpression of the hsp60 in cytosolic soluble proteins and membrane pellets in cisplatin-resistant cell lines, demonstrating a substantial increase in hsp60 in cytosolic and membrane pellet fractions in 7404-CP20 cells. Overexpression of hsp60 could also be detected in the KB-CP20 cells (about 2-fold higher than the parental KB-3-1 cells). hsp70 levels (lower panels) were generally unchanged in cisplatin-resistant cells and their parental cells for both hepatoma and KB cell lines.

### Discussion

Several different mechanisms for resistance to cisplatin have been demonstrated during the past several years, including decreased cross-linking of DNA, increased rates of DNA repair (Chu and Chang, 1990), increased levels of intracellular thiols and reduced accumulation of cisplatin (Richon *et al.*, 1987; Andrews and Howell, 1990). However, no one mechanism has been uniformly present in all cisplatin-resistant cells examined. In this work, we find that two human cell lines selected for high-level resistance to cisplatin, which also show similar patterns of cross-resistance to methotrexate, 5-FU and 6-MP, manifest many changes in protein levels, but share an increase in polypeptides of molecular weight 52 and 50 kDa.

The human liver carcinoma cell line BEL-7404 was chosen as a model system to study cisplatin resistance because of its intrinsic resistance to this agent and in the hope that further selection in cisplatin would amplify the effect of its intrinsic resistance mechanism. Thus, it should be feasible to determine which biochemical alterations are responsible for the intrinsic and acquired cisplatin resistance of these cells with the central goal of creating sensitive molecular probes of clinical tumour specimens. A comparison of cisplatin resistance between the BEL-7404 hepatoma cells and KB-3-1 adenocarcinoma cells showed that the hepatoma cells were about 13-fold more resistant, without selection, than the KB-3-1 cells. After selection in cisplatin, the $IC_{50}$ values for the 7404-CP7.5 cells and the KB-CP10 cells were 49 and 38 $\mu g\ ml^{-1}$ cisplatin respectively, which was almost one thousand times higher than the parental KB-3-1 cells. We would like to determine the basis for such high-level resistance to cisplatin, which is not commonly seen in cell lines selected *in vitro* for resistance to this agent.

It has been reported that cells acquiring cisplatin resistance also develop resistance to alkylating agents and other types of DNA-damaging chemicals (Frei *et al.*, 1985). 7404-CP$^r$ cells showed a limited degree of resistance to a bifunctional agent, melphalan (2.6-fold), that was similar to results previously described (Puchalski and Fahl, 1990), while the KB-CP10 cells were about 11-fold more resistant than the parental KB-3-1 cells. Interestingly, both 7404-CP$^r$ and KB-CP$^r$ cells demonstrated cross-resistance to methotrexate, about 39- and 20-fold relative to their sensitive parental cell

**Figure 7** Fluorogram of two-dimensional gels of [$^{35}$S]methionine-labelled membrane proteins. (**a**) BEL-7404; (**b**) 7404-CP20; (**c**) KB-3-1; and (**d**) KB-CP20. Cell membrane fractions were prepared as described in Materials and methods. Arrows labelled with I or R were positioned as described in Figure 6.

**Figure 8** Immunoblot analysis of heat shock proteins with visualisation by ECL. Cytosolic soluble proteins (**a**) and membrane pellet fractions (**b**) were transblotted on nitrocellulose membrane after 10% SDS-PAGE as described in Materials and methods. Top: Protein samples were immunoreacted with monoclonal antibody (MAb) specific to human hsp60 (arrow). Bottom: The same blot in upper panel was stripped and reprobed with MAb specific to human hsp70 (arrow). Lane 1, BEL-7404; lane 2, 7404-CP20; lane 3, KB-3-1; lane 4, KB-CP20.

lines respectively. This phenomenon is consistent with the observations on cisplatin-resistant cell lines derived from a human squamous carcinoma, SSC-25 (Teicher *et al.*, 1986), and an ovarian carcinoma (Newman *et al.*, 1988). In a recent study on mouse Balb/3T3 cisplatin-resistant cell lines transformed with human genomic DNAs isolated from human hepatoma 7404-CP7.5 cells, cross-resistance to methotrexate also occurred (unpublished data of the authors). The human cisplatin-resistant hepatoma cells also showed cross-resistance to 5-fluorouracil and 6-mercaptopurine by 23.0- and 12.9-fold relative to the parental cells respectively. Similar patterns but lower resistance levels to 5-fluorouracil and 6-mercaptopurine were also found in the KB-CP10 cells.

These results suggest that this cross-resistance, particularly to methotrexate, may be a consequence of a common, novel mechanism of multidrug resistance. This cross-resistance may be related to nucleotide pools since cross-resistance primarily affects nucleotide derivatives, or could be associated with export of drug metabolites via an ATP-dependent glutathione *S*-conjugate export pump (Ellis, 1990; Ishikawa and Ali-Osman, 1993). More recent data from collaborative studies with Dr Thomas C Hamilton at Fox Chase Cancer Center indicates that there is a 17-fold reduction in accumulation of cisplatin in the cisplatin-resistant hepatoma 7404-CP20 cells compared with the parental sensitive cell line BEL-7404 (SW Johnson and TC Hamilton, personal communication). This result suggests that an active efflux pump or an impaired uptake of cisplatin may exist in these cisplatin-resistant cells (Jekunen *et al.*, 1994).

No cross-resistance to the MDR substrates colchicine, vinblastine, actinomycin D, mitomycin C and VP-16, or to non-MDR substrate hydroxyurea was found in the hepatoma CP7.5 cells. Some cross-resistance to actinomycin D, doxorubicin, mitomycin C and hydroxyurea was observed in the KB-CP10 cells. These results support the hypothesis that there is no single pattern of resistance associated with selection in cisplatin and that the multiple steps of selection used in this study undoubtedly gave rise to several different resistance mechanisms.

To begin our analysis of the basis of drug resistance in these highly cisplatin-resistant human cell lines, we compared patterns of protein expression seen in KB-CP10 and CP7.5 cells. Alterations in the steady-state amount of several different polypeptides were repeatedly detected in both soluble cytosolic and crude membrane fractions by regular SDS-

PAGE or two-dimensional SDS–PAGE. When comparing the differences between the cisplatin-resistant cells and their sensitive parental cells, one feature common to 7404-CP$^r$ and KB-CP$^r$ was the overexpression of a 52 kDa protein(s) in the pelleted membrane and cytosolic fractions of both the hepatoma and the KB cisplatin-resistant cell lines. The content of this protein(s) in crude membrane fractions increased in parallel with increased cisplatin resistance in the 7404-CP$^r$ cell lines, and reduced amounts were found in the revertant KB-CP5-df365 cells which had been maintained in cisplatin-free medium for more than 1 year. The resistance of this KB-CP5-df365 cell line to cisplatin was remarkably reduced from its original 788 times to 21 times higher than the sensitive parental KB-3-1 cells. However, the 52 kDa protein(s) was still increased to some extent in a partially reverted cisplatin-resistant hepatoma cell line, 7404-CP20-df155, which retained 31-fold more resistance to cisplatin than its sensitive parental cells. This continued expression of cisplatin resistance and of 52 kDa protein(s) indicates that cisplatin resistance is a relatively stable change in cells, and that the increase in the 52 kDa protein(s) is also probably a stable change rather than a transient event such as a toxic response to cisplatin. Further evidence that the 52 kDa protein is not increased directly in response to cisplatin treatment came from an experiment in which the sensitive parental BEL-7404 cells were exposed to cisplatin at 1.5 $\mu g\ ml^{-1}$ for 1, 3, and 17 h (data not shown). In this experiment, no increase in 52 kDa proteins was observed. Compared with KB-CP10 cells, elevations and reductions in several other proteins of different molecular weight were also observed, but no other changes were shared by both the hepatoma and the KB cell lines.

Recent studies indicate that 52 kDa protein(s) can also be detected in primary and secondary transfectants of mouse Balb/3T3 cells transformed to express cisplatin resistance with high molecular weight DNA from cisplatin-resistant human hepatoma 7404-CP7.5 cells, suggesting that the 52 kDa proteins may play a role, directly or indirectly, in cisplatin resistance (DW Shen, I Pastan and MM Gottesman, in preparation). Amino acid microsequencing of the 52 kDa spots indicates that there is 100% identity for seven amino acids to the human heat shock protein, hhsp60, a chaperonin. Using immunoblots reacted with a human specific monoclonal antibody directed to hsp60 further confirmed that heat shock protein 60 was elevated in CP$^r$ cells from both hepatoma and KB cell lines. There were no detectable changes in hsp70 between sensitive and CP$^r$ cell lines tested in this study. Work by Howell's group has also identified hsp60 as a protein overexpressed in cisplatin-resistant cells (Kimura *et al.*, 1993). At this time, we can only speculate on the possible functions of an elevation in hsp60 in cisplatin-resistant human cancer cells. hsp60 is a homologue of GroEL and a highly conserved intrinsic mitochondrial protein. As a member of the chaperonin family, it is generally accepted that the hsp60 probably mediates the correct folding of polypeptides, and in some cases their assembly into oligomeric structures. hsp60 may also function by binding specifically and non-covalently to interactive protein surfaces that are exposed transiently during cellular processes such as protein synthesis, protein transport across membranes and stress responses (Ellis, 1990). Whether overexpression of hsp60 protects cells from toxic effects of cisplatin is as yet unclear.

Of the other protein changes, only the overexpressed I-90 protein in the soluble fractions of cisplatin-resistant cells could be tentatively identified as a stress-related heat shock protein, hsp90, as its isoelectric point (Figure 6b) is quite similar to that reported for hsp90 on the two-dimensional map of transformed amnion cells (Celis *et al.*, 1990). The altered expression of other proteins, in either 7404 or KB cisplatin-resistant cells, demonstrates that profound phenotypic alterations occurred during acquisition of resistance to the agent. However, the changes in these proteins may not be directly responsible for resistance to cisplatin, but may reflect a response to stress and/or represent changes needed for survival and proliferation of cells exposed to cisplatin.

In these studies, the 200 kDa protein found in cisplatin-resistant murine lymphoma cells (Kawai *et al.*, 1990) was not detected in our CP$^r$ cell lines by SDS–PAGE. In addition, no elevated expression of P-glycoprotein, the *MDR*1 gene product, was found as compared with KB-C1.5, an *MDR*1 gene-expressing cell line. Expression of the *MDR*1 gene was undetectable by Northern or RNA slot-blot hybridisation with the specific *MDR*1 cDNA probe pHDR5A (Ueda *et al.*, 1987 and results not shown). Neither extrachromosomal DNA (episomes) nor double minute (DM) chromosomes could be identified by pulsed-field gradient gel electrophoresis (PFGE) or karyotypic analysis. In addition, the native *MDR* locus, which is contained within a 330 kb *Sfi*I fragment, was intact and unamplified as determined by PFGE analysis (Schoenlein *et al.*, 1992, and data not shown).

Recently, changes in DNA damage repair proteins, DNA-binding proteins, chromosomal protein HMG1 and nuclear matrix proteins were found in cisplatin-resistant cells (Chu and Chang, 1990; Clugston *et al.*, 1992; de Jong *et al.*, 1992; Pil and Lippard, 1992; Zhen *et al.*, 1992). Some types of glutathione *S*-transferase, glutamylcystine synthetase and thymidylate synthetase have also been reported to increase during development of cisplatin resistance (Behrens *et al.*, 1987; Scanlon and Kashani-Sabet, 1988; Puchalski and Fahl, 1990; Godwin *et al.*, 1992). Taken together with the results in this paper, cisplatin resistance appears to result from many different mechanisms affecting function of the nucleus, cytoplasm and membranes. Which of these mechanisms is most common and which, if any, are clinically relevant, remains to be determined.

**Abbreviations:** cisplatin, *cis*-diamminedichloroplatinum(II); CP$^r$, cisplatin resistance; SDS–PAGE, sodium dodecyl sulphate–polyacrylamide gel electrophoresis; DMS, double minutes (chromosomes); EMS, ethyl methanesulphonate.

**Acknowledgements**
We would like to thank Bristol-Myers Research Laboratory and Johnson Matthey for their gifts of cisplatin, Drs Nan Wang and John Barrett for useful discussions, Cathy Changchien for technical assistance, and Althea Jackson and Paula Morgan for secretarial assistance.

### References

ANDREWS PA AND HOWELL SB. (1990). Cellular pharmacology of cisplatin: perspectives on mechanisms of acquired resistance. *Cancer Cells*, **2**, 35–43.

BEHRENS BC, HAMILTON TC AND OZOLS RF. (1987). Characterization of a *cis*-diamminedichloroplatinum(II)-resistant human ovarian cancer cell line and its use in evaluation of platinum analogues. *Cancer Res.*, **47**, 414–418.

CELIS JE, GESSER B, RASMUSSEN HH, MADSEN P, LEFFERS H, DEJGAARD K, HONORE B, OLSEN E, RATZ G, LAURIDSEN JB, BASSE B, MOURITZEN S, HELLERUP M, ANDERSEN A, WALBUM E, CELIS A, BAUW G, PUYPE M, DAMME JV AND VANDEKERCKHOVE J. (1990). Comprehensive two-dimensional gel protein database offer a global approach to the analysis of human cells: the transformed amnion cells (AMA) master database and its link to genome DNA sequence data. *Electrophoresis*, **11**, 989–1071.

CHU G AND CHANG E. (1990). Cisplatin-resistant cells express increased levels of a factor that recognizes damaged DNA. *Proc. Natl Acad. Sci. USA*, **87**, 3324–3327.

CLUGSTON CK, MCLAUGHLIN K, KENNY MK AND BROWN R. (1992). Binding of human single-stranded DNA binding protein to DNA damaged by the anticancer drug cis-diamminedichloroplatinum(II). *Cancer Res.*, **52**, 6375–6379.

DE JONG S, TIMMER-BOSSCHA H, DE VRIES EGE AND MULDER NH. (1992). Effect of novobiocin on cisplatin cytotoxicity and DNA interstrand cross-link formation in a cisplatin-resistant, small-cell lung carcinoma cell line. *Int. J. Cancer*, **53**, 110–117.

ELLIS RJ. (1990). The molecular chaperone concept. *Cell Biol.*, **1**, 1–9.

FREI E, CUCCHI CA AND ROSOWSKY A. (1985). Alkylating agent resistance: in vitro studies with human cell lines. *Proc. Natl Acad. USA*, **82**, 2158–2162.

GODWIN AK, MEISTER A AND ANDERSON ME. (1992). High resistance to cisplatin in human ovarian cancer cell lines is associated with marked increase of glutathione synthesis. *Proc. Natl Acad. Sci. USA*, **89**, 3070–3074.

GOTTESMAN MM AND PASTAN I. (1993). Biochemistry of multidrug resistance mediated by the multidrug transporter. *Annu. Rev. Biochem.*, **62**, 385–427.

ISHIKAWA T AND ALI-OSMAN F. (1993). Glutathione-associated cis-diamminedichloroplatinum(II) metabolism and ATP-dependent efflux from leukemia cells. *J. Biol. Chem.*, **268**, 20116–20125.

JEKUNEN AP, HOM DK, ALCARAZ JE, EASTMAN A AND HOWELL SB. (1994). Cellular pharmacology of dichloro(ethylenediamine) platinum(II) in cisplatin-sensitive and resistant human ovarian carcinoma cells. *Cancer Res.*, **54**, 2680–2687.

KASAHRA K, FUJIWARA YA AND NISHIO K. (1991). Metallothionein content correlates with the sensitivity of human small cell lung cancer cell lines to cisplatin. *Cancer Res.*, **51**, 3237–3242.

KAWAI K, KAMATANI N, GEORGES E AND LING V. (1990). Identification of a membrane glycoprotein overexpressed in murine lymphoma sublines resistant to *cis*-diamminedichloroplatinum(II). *J. Biol. Chem.*, **265**, 13137–13142.

KIMURA E, ENNS RE, THIEBAUT R AND HOWELL SB. (1993). Regulation of HSP60 mRNA expression in a human ovarian carcinoma cell line. *Cancer Chemother. Pharmacol.*, **32**, 279–285.

LAEMMLI UK. (1970). Cleavage of structural proteins during the assembly of the head of bacteriophage T4. *Nature*, **227**, 680–685.

LOEHRER PJ AND EINHORN LH. (1984). Cisplatin. *Ann. Intern. Med.*, **100**, 704–713.

MASUDA H, OZOLS RF, LAI G, FOJO A, ROTHENBERG M AND HAMILTON TC. (1988). Increased DNA repair as a mechanism of acquired resistance to *cis*-diamminedichloroplatinum(II) in human ovarian cancer cell lines. *Cancer Res.*, **48**, 5713–5716.

MOSCOW JA AND COWAN KH. (1988). Multidrug resistance. *J. Natl Cancer Inst.*, **80**, 14–20.

NEWMAN EM, LU Y, KASHANI-SABET M, KESAVAN V AND SCANLON KJ. (1988). Mechanisms of cross-resistance to methotrexate and 5-fluorouracil in an A2780 human ovarian carcinoma cell subline resistant to cisplatin. *Biochem. Pharmacol.*, **37**, 443–447.

O'FARRELL PZ, GOODMAN HM AND O'FARRELL PH. (1977). High resolution two-dimensional electrophoresis of basic as well as acidic proteins. *Cell*, **12**, 1133–1142.

PIL PM AND LIPPARD SJ. (1992). Specific binding of chromosomal protein HMG1 to DNA damaged by the anticancer drug cisplatin. *Science*, **256**, 234–237.

PLOOY ACM, VAN DIJK M, BERENDS F AND LOHMAN PHM. (1985). Formation and repair of interstrand cross-links in relation to cytotoxicity and unscheduled DNA synthesis induced in control and mutant human cells treated with *cis*-diamminedichloroplatinum (II). *Cancer Res.*, **45**, 4178–4184.

PUCHALSKI RB AND FAHL WE. (1990). Expression of recombinant glutathione-S-transferase pi, Ya, or Yb1 confers resistance to alkylating agents. *Proc. Natl Acad. Sci. USA*, **87**, 2443–2447.

RICHON VM, SCHULTE N AND EASTMAN A. (1987). Multiple mechanisms of resistance to *cis*-diamminedichloroplatinum(II) in murine leukemia L1210 cells. *Cancer Res.*, **47**, 2056–2061.

SCANLON KJ AND KASHANI-SABET M. (1988). Elevated expression of thymidylate synthase cycle genes in cisplatin-resistant human ovarian carcinoma A2780 cells. *Proc. Natl Acad. Sci. USA*, **85**, 650–653.

SCHOENLEIN PV, SHEN DW, BARRETT JT, PASTAN I AND GOTTESMAN MM. (1992). Double minute chromosomes carrying the human multidrug resistance 1 and 2 gene are generated from the dimerization of submicroscopic circular DNAs in colchicine-selected KB carcinoma cells. *Mol. Biol. Cell.*, **3**, 507–520.

SHELLARD SA, HOSKING LK AND HILL BT. (1991). Anomalous relationship between cisplatin sensitivity and the formation and removal of platinum-DNA adducts in two human ovarian carcinoma cell lines *in vitro*. *Cancer Res.*, **51**, 4557–4564.

SHEN DW AND CHEN JM. (1985). Studies on human hepatocellular carcinoma cells cultured *in vitro*. In *Subclinical Hepatocellular Carcinoma*, Tang ZY (ed.) pp. 336–346, Springer: New York.

SHEN DW, FOJO A, CHIN JE, RONINSON IB, RICHERT N, PASTAN I AND GOTTESMAN MM. (1986). Human multidrug-resistant cell lines: increased mdr1 expression can precede gene amplification. *Science*, **232**, 643–645.

SHEN DW, LU Y, CHIN KV, PASTAN I AND GOTTESMAN MM. (1991). Human hepatocellular carcinoma cell lines exhibit multidrug resistance unrelated to *MDR*1 gene expression. *J. Cell Sci.*, **98**, 317–322.

TEICHER BA, CUCCHI CA, LEE JB, FLATOW JL, ROSOWSKY A AND FREI E. (1986). III, Alkylating agents: in vitro studies of cross-resistance patterns in human tumour cell lines. *Cancer Res.*, **46**, 4379–4383.

TIMMER-BOSSCHA H, MULDER NH AND DE VRIES EGE. (1992). Modulation of *cis*-diamminedichloroplatinum(II) resistance: a review. *Br. J. Cancer*, **66**, 227–238.

UEDA K, CLARK DP, CHEN C, RONINSON I, GOTTESMAN MM AND PASTAN I. (1987). The human multidrug resistance (*mdr*1) gene. *J. Biol. Chem.*, **262**, 505–508.

ZHEN W, LINK CJ, O'CONNOR PM, REED E, PARKER R, HOWELL WB AND BOHOR VA. (1992). Increased gene-specific repair of cisplatin interstrand cross-links in cisplatin-resistant human ovarian cancer cell lines. *Mol. Cell. Biol.*, **12**, 3689–3698.